# *CYP2C19* pharmacogenetics in advanced cancer: compromised function independent of genotype

**DOI:** 10.1038/sj.bjc.6604699

**Published:** 2008-10-14

**Authors:** N A Helsby, W-Y Lo, K Sharples, G Riley, M Murray, K Spells, M Dzhelai, A Simpson, M Findlay

**Affiliations:** 1 Department of Molecular Medicine and Pathology, University of Auckland, Auckland, New Zealand; 2 Auckland Cancer Society Research Centre, University of Auckland, Auckland, New Zealand; 3 Cancer Trials New Zealand, University of Auckland, Auckland, New Zealand; 4 Department of Preventive and Social Medicine, University of Otago, Dunedin, Otago, New Zealand; 5 Regional Cancer Centre, Auckland District Health Board, Auckland, New Zealand; 6 Wellington Cancer Centre, Capital and Coast District Health Board, Wellington, New Zealand

**Keywords:** loss of function, pharmacogenetics, CYP2C19, advanced cancer, drug metabolism, inflammation

## Abstract

CYP2C19 is a drug-metabolising enzyme involved in the metabolism of a number of chemotherapeutic agents including cyclophosphamide. Variants of the *CYP2C19* gene result in a loss of function polymorphism, which affects approximately 3% of the Caucasian population. These individuals are poor metabolisers (PM) of a wide range of medications including omeprazole (OMP). In healthy subjects PM can be identified through homozygous variant genotype. However, a discordance between *CYP2C19* genotype and phenotype has been reported previously in a small study of cancer patients. To investigate whether CYP2C19 activity was decreased in patients with advanced cancer, *CYP2C19* genotype was determined in 33 advanced cancer patients using PCR-RFLP analysis for the two important allelic variants (*2,681G>A and *3,636G>A) and the activity of the enzyme was evaluated using the CYP2C19 probe drug OMP. The activity of the drug-metabolising enzyme CYP2C19 was severely compromised in advanced cancer patients, resulting in a PM status in 37% of the patients who had normal genotype. This is significantly (*P*<0.0005) higher than that would be predicted from the genotypic status of these patients. There was no evidence of a correlation between compromised CYP2C19 activity and any of the proinflammatory cytokines or acute phase response proteins studied. However, there was preliminary evidence of an association between PM status and low body mass (*P*=0.03). There is increasing interest in using pharmacogenetics to ‘individualise medicine’, however, the results of this study indicate that in a cancer population genotyping for *CYP2C19* would significantly underestimate the number of phenotypic PM of drugs, such as cyclophosphamide, which may be metabolised by this enzyme.

Variation in drug disposition and response is associated with many anticancer agents, in part due to large inter-patient differences in pharmacokinetics (Petros and Evans, 2004). This may be the result of variable expression of hepatic cytochrome P450 (CYP), enzymes which play an important role in the metabolism and elimination of many chemotherapeutic drugs.

CYP2C19 is an important member of the CYP family of enzymes and is involved in the metabolism of a diverse range of drug substrates such as the antiulcer agent omeprazole (OMP) (Desta *et al*, 2002) and the antimalarial drug proguanil (Ward *et al*, 1991). Recently, it has become apparent that CYP2C19 is also important in the disposition of a number of chemotherapeutic agents, including cyclophosphamide (Takada *et al*, 2004; Timm *et al*, 2005), thalidomide (Ando *et al*, 2002; Li *et al*, 2007) and bortezomib (Uttamsingh *et al*, 2005).

There is a loss of function genetic polymorphism associated with the CYP2C19 enzyme, with the result that individuals who are homozygous variant (*var*/*var*) are ‘poor metabolisers’ (PM) of drugs that are substrates for CYP2C19 (Desta *et al*, 2002). Approximately 3% of Caucasians and up to 30% of Asians are *CYP2C19* PM (Xie *et al*, 1999). Numerous studies have shown a correlation between variant genotype and PM phenotype in healthy populations (Xie *et al*, 1999; Desta *et al*, 2002). Hence, the use of a genotyping test for *CYP2C19* metaboliser status is often advocated as a substitute for direct phenotyping with validated probe drugs such as OMP.

However, genetic variability is not the only factor that can affect functional activity of drug-metabolising enzymes; gender, age and co-medications can all play a role in variable CYP expression. In particular, co-medications can mimic the genetic variability observed for CYP2C19 with the result that a patient can become a PM ‘phenocopy’ due to inhibition (competition) of drug metabolism. However, the importance of chronic disease as a contributor to variable drug disposition is often overlooked.

There is increasing evidence that the activity of CYP is decreased in cancer. In a small study of patients (*n*=16) with advanced cancer (Williams *et al*, 2000), all individuals had normal *CYP2C19* genotype but 25% of these patients were CYP2C19 PM phenocopies. However, the mechanism behind this genotype–phenotype discordance was not investigated. Previous studies have demonstrated that the activity of a different enzyme, CYP3A4, is also decreased in advanced cancer patients and that the decreased activity correlates with inflammatory response (Rivory *et al*, 2002). An inverse relationship between plasma cytokine (TNF-*α* and IL-6) concentrations and CYP2C19 activity has been demonstrated in patients with congestive heart failure (Frye *et al*, 2002). Moreover, *CYP2C19* mRNA expression is reported to be downregulated by cytokines in human hepatocyte *in vitro* cultures (Aitken and Morgan, 2007). Hence, limited evidence indicates that advanced cancer patients may have decreased capacity to metabolise certain chemotherapeutic agents due to decreased CYP activity.

The aim of this study was to confirm whether or not CYP2C19 activity is decreased in patients with advanced cancer and to determine whether a relationship exists between CYP2C19 metaboliser status and inflammatory markers such as cytokines and acute phase response proteins.

## Materials and methods

This study (NTX/05/07/083) was approved by the New Zealand Health and Disability Multi-Regional Ethics Committee. Following full informed written consent, 33 patients (18 male, 15 female) with advanced cancer were recruited into the study. All patients were self-reported as Caucasian with the exception of one patient who was Asian. Patients had not received any chemotherapy for at least 4 weeks before the study and were not on any medications that are known inducers or inhibitors of CYP2C19. All patients had adequate hepatic and renal function (creatinine levels <0.12 mmol l^−1^, AST/ALT <90 U l^−1^, ALP <300 U l^−1^, bilirubin <20 *μ*mol l^−1^).

Blood (8.5 ml) was collected in PAXgene blood tubes and DNA was extracted using the PAXgene™ blood DNA kit (Qiagen, Hilden, Germany). The *CYP2C19* genotype was determined by PCR-RFLP analysis of the two important allelic variants (CYP2C19*2 and CYP2C19*3) using previously published methods (Goldstein and Blaisdell, 1996).

The activity of CYP2C19 (phenotype) was determined using OMP as the probe substrate (Balian *et al*, 1995). The plasma concentration of OMP and the 5′-hydroxy metabolite (5′-OH) were measured in the patient's plasma 2 h after oral administration of OMP (20 mg) using previously published methods (Woolf and Matuszewski, 1998; Motevalian *et al*, 1999; Orlando and Bonato, 2003) with slight alterations. The log OMP hydroxylation index (log_10_ [OMP/5′OH]) was used to describe the CYP2C19 metabolic status of the patients. An index of <1 has been previously shown to correspond to an extensive metaboliser (EM) phenotype and an index >1 is a phenotypic PM (Balian *et al*, 1995; Kortunay *et al*, 1997).

Serum cytokine levels (IL-1*α*, IL-1*β*, IL-6, TNF-*α*, TGF-*β*) were measured by Lincoplex® multiplex immunoassay (LINCO Research, St Charles, MO, USA). The levels of hsCRP were determined by the local medical laboratory testing service using the standard nephelometry assay. The levels of growth hormone and albumin were determined using the commercially available ELISA kits (Growth hormone: R&D Systems, Minneapolis, MN, USA; Albumin: BioAssay Systems, Hayward, CA, USA).

Statistical analyses were determined using Stata Statistical Software (Release 9, StataCorp LP) and R (R Development Core Team, 2007). Continuous data are reported as median and interquartile range (IQR). Associations between continuous variables were assessed using Spearman's rank correlation, and Mann–Whitney rank-sum tests were used to compare continuous variables across two groups. The discordance between *CYP2C19* genotype *vs* phenotype was assessed using McNemar's test and proportions were compared using Fisher's exact test. Two-sided *P-*values <0.05 were considered statistically significant.

## Results

All patients in the study had advanced incurable cancer and a range of primary tumours including breast, lung, gastrointestinal tract, kidney, ovarian and melanoma. Patients were aged between 33 and 79 years, with a mean age of 61.5 years and there were 18 male and 15 female patients in the study.

The *CYP2C19* genotype of the patients was determined following PCR-RFLP analysis of the allelic variants (CYP2C19*2 and CYP2C19*3). Previous studies have shown that individuals who are homozygous variant (*var*/*var*) for the *CYP2C19* gene have null CYP2C19 activity and are PM. In contrast, individuals with at least one wild-type allele at these loci are classed as EM, that is, *wt*/*wt* and *wt*/*var.* One patient was homozygous variant (*var*/*var*) for the CYP2C19*3 allele; hence, they are a genotypic PM (gPM) and would be predicted to have null CYP2C19 activity. Genetic PM are more common in Asian populations, and this was the one Asian patient in the study. The CYP2C19*3 allele was not observed in any other patient; however, this variant is uncommon in Caucasian populations. The remaining patients were *wt*/*wt* (*n*=20) or *wt/var* (*n*=12) for the CYP2C19*2 allele and hence are all classed as genotypic EMs.

**Figure 1:**
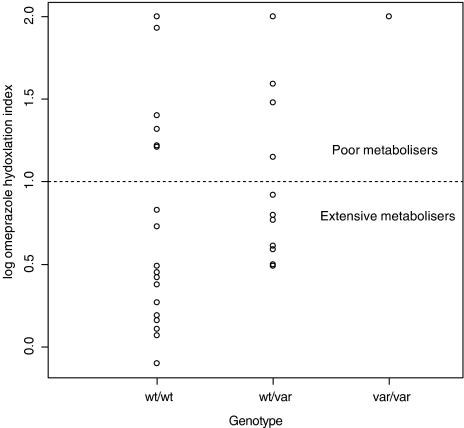
Distribution of CYP2C19 activity in patients with advanced cancer. Omeprazole (OMP) was used as a probe substrate for the CYP2C19 enzyme and activity was determined using the log OMP hydroxylation index (log OMP HI) at 2 h post-dose. Individuals with a log OMP HI >1 are classed as phenotypic poor metabolisers of this drug.

The activity of the CYP2C19 enzyme in the patients was then determined directly using OMP as a probe drug. Two patients had no detectable drug or metabolite in the plasma and were excluded from any further analysis. The medians of the log OMP hydroxylation index were 0.86 and 0.47 for the *wt*/*var* and *wt*/*wt* genotypes, respectively (data shown in Figure 1). The one genotypic PM (*var*/*var*) in this study had a log OMP hydroxylation index of 2. This is consistent with the predicted null CYP2C19 activity in this patient. Comparison of the genotype predicted and the measured metabolic status (Table 1) found a statistically significant discordance (*P*<0.0005). Of the 30 cancer patients with genotypic EM status 11 (37%) were CYP2C19 PM ‘phenocopies’; there was no statistically significant difference between the proportions who were phenotypic PM in the two genotypic EM groups (6 out of 18 for *wt*/*wt* and 5 out of 12 for *wt*/*var*, *P*=0.6). Five patients with *wt* alleles had liver metastases and the hydroxylation index in these patients were 0.11, 0.59, 1.22, 1.32 and 1.48, that is, two patients were phenotypic EM and three were phenotypic PM.

**Figure 2:**
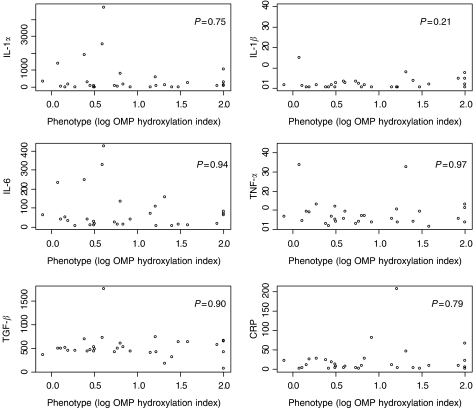
Relationship between CYP2C19 activity and the levels of inflammatory cytokines and CRP. The *P*-values from Spearman's rank correlation test are shown on the graphs.

A number of studies have demonstrated that acute inflammation can downregulate P450 enzyme expression, (reviewed by Morgan *et al*, 2008). To determine whether the decreased CYP2C19 activity observed in the cancer patients was related to the inflammatory response, the serum levels of a number of proinflammatory cytokines and the acute phase response protein, CRP were measured. There was considerable variability in the levels of IL-1*α*, IL-1*β*, IL-6, TNF-*α* and TGF-*β* and these were clearly elevated in some patients, with a median (IQR) for IL-1*α* of 107.2 (19.8–408.2); IL-1*β*, 1.1 (0–2.7); IL-6, 40.0 (13.3–89.1); TNF-*α*, 6.5 (4.0–10.8) and TGF-*β*, 495.0 (427.5–627.5) pg ml^−1^. The acute phase protein, hs-CRP, was above the upper limit of normal (10 mg l^−1^) in 15 patients; however, only five individuals had levels >40 mg l^−1^. There was no statistically significant correlation between the levels of any individual cytokine and CYP2C19 metabolic activity (log OMP hydroxylation index) (Figure 2, Table 2). Multiple regression analysis also did not reveal any evidence of correlation between inflammatory markers and CYP2C19 activity in the patients. The acute phase inflammatory response also results in hypoalbuminaemia and this has been associated with decreased activity of other CYP enzymes in cancer patients (Rivory *et al*, 2002). The median albumin concentration in this study was 45.4 g l^−1^ (IQR 43.7–48.8) and only one patient was hypoalbuminaemic (below 35 g l^−1^). However, there was no evidence of association between albumin concentrations and log OMP hydroxylation index in the patients (Table 2).

Owing to the lack of association between proinflammatory cytokines and CYP2C19 activity in the advanced cancer patients, the samples were also analysed for the levels of growth hormone. Administration of growth hormone has been associated with a decrease in OMP hydroxylation index in healthy volunteers (Jürgens *et al*, 2002). In addition, growth hormone has been reported to be elevated in cancer patients (Dülger *et al*, 2004). There was considerable variability in the levels of growth hormone in the cancer patients (median=0.9, IQR=0.3–3.0 *μ*g l^−1^); however, all values were within expected daily variation of this hormone and no patients had levels elevated above normal values (>24 *μ*g l^−1^). Moreover, there was no evidence of association between the levels of growth hormone and CYP2C19 activity (log OMP hydroxylation index) in the cancer patients (Table 2).

Another indicator of disease progression in cancer is cachexia, particularly skeletal muscle loss. Although this study did not directly assess the patients’ cachexic status, body mass index (BMI) was determined in these patients at the time of recruitment. There was preliminary evidence of an association between BMI and CYP2C19 activity in the advanced cancer patients; according to the standard cutoff for BMI, among those with low BMI (<25) 62% (8 of 13) were PM compared with 22% (4 of 18) in patients with a high BMI (>25) (*P*=0.03).

## Discussion

The results of this study indicate that the activity of the drug-metabolising enzyme CYP2C19 was compromised in advanced cancer patients. Of the advanced cancer patients studied who had genotypic EM status, 37% were PM phenocopies. In healthy subjects, only 3% of Caucasian subjects are CYP2C19 PM and this is due to the presence of the *CYP2C19* loss of function genotype (homozygous variant) in these individuals (Xie *et al*, 1999).

In a healthy population, the activity of CYP2C19 is bimodally distributed and the antimode of the population (log OMP hydroxylation index=1.0) can delineate between *CYP2C19* genotypic EM (*wt*/*wt* and *wt*/*var*) and genotypic PM (*var*/*var*) genotypes. The log OMP hydroxylation index in genotypic EM is reported to be between −0.55 and 0.40 (Balian *et al*, 1995). In contrast, the activity of the enzyme in the advanced cancer population was not bimodally distributed and the activity of the enzyme was low (log OMP hydroxylation index range −0.10 to 2.0) indicating that the overall activity of this enzyme was compromised in this cancer population.

This is not the first study to demonstrate the effect of advanced cancer on drug-metabolising enzymes in advanced cancer. A discordance between CYP2C19 genotype and phenotype has been reported previously in a small study (*n*=16) of advanced cancer patients (Williams *et al*, 2000). The higher incidence of phenotypic PM in this study (37%) compared with an incidence of 25% (Williams *et al*, 2000) may reflect small sample variation. However, the high incidence of PM in both studies indicates that decreased CYP2C19 activity in cancer patients is a noteworthy effect that could have clinical significance for chemotherapeutics, such as cyclophosphamide, which may be substrates for CYP2C19.

A number of clinical factors may influence the activity of hepatic enzymes, including co-medications and liver disease. However, no patients in this study had taken any medications that could induce or inhibit CYP2C19 and patients had not received any cancer chemotherapy for at least 4 weeks before the study. Inclusion criteria for the study also required patients to have good hepatic and renal function, in contrast to the previous small study (Williams *et al*, 2000) in which a number of patients had elevated ALP enzyme and two patients had hepatitis. Although five patients in this study had liver metastases, this did not appear to correlate with compromised CYP2C19 metaboliser status and there was also no relationship with the location of the primary cancer and metaboliser status.

An additional factor hypothesised to affect the activity of hepatic CYP enzymes is inflammation. The activity of another hepatic drug-metabolising enzyme CYP3A is decreased in cancer patients (Rivory *et al*, 2002) and this decreased activity correlates with the acute phase response status of the patients. In addition, an inverse relationship between CYP2C19 activity and plasma TNF-*α* or IL-6 concentrations has been reported in patients with congestive heart failure (Frye *et al*, 2002). A more recent study (Aitken and Morgan, 2007) using cultured human hepatocytes has reported that a number of cytokines can downregulate *CYP2C19* mRNA expression *in vitro*. However, in this study in advanced cancer patients, we could not find evidence of a relationship between CYP2C19 activity and serum levels of cytokines or the acute phase response protein CRP. Nevertheless, alternate cytokines or other inflammatory mediators may be involved in the observed decreased CYP2C19 function in advanced cancer.

The levels of cytokines observed in our study were in the same range as the data reported previously in cancer patients (Rivory *et al*, 2002) and in patients with congestive heart failure (Frye *et al*, 2002). But, it should be noted that the levels (pg ml^−1^) of the cytokines observed in cancer patients in this study is approximately 100-fold lower than the 10 ng ml^−1^ concentrations of IL-6 and TGF-*β* required to significantly downregulate *CYP2C19* mRNA *in vitro* (Aitken and Morgan, 2007). However, the circulating half-life of cytokines is relatively short and the levels may also be underestimated due to the presence of soluble receptors for these inflammatory mediators. Moreover, little is known about the temporal relationship between circulating cytokine levels and downregulation of CYP *in vivo*.

Further studies in larger cohorts of patients are required to explore these hypotheses more fully.

We also did not observe a relationship between decreased CYP2C19 activity and the acute phase response protein CRP. This may again be due to the relatively low levels of this inflammatory marker observed in the patients. The relationship between CRP levels and decreased CYP3A activity demonstrated previously (Rivory *et al*, 2002), occurred at relatively high concentrations of CRP, in contrast only five patients in this study had CRP levels >40 mg l^−1^. The levels >40 mg ml^−1^ are considered to be indicative of acute inflammation (Gill, 2000). Hence, the inflammatory mediators in this population of advanced cancer patients may not have achieved levels high enough to have an effect on CYP2C19 activity.

Administration of growth hormone can decrease CYP2C19 activity in man (Jürgens *et al*, 2002). In addition, growth hormone has been reported to be elevated in cancer patients (Dülger *et al*, 2004). However, there also did not appear to be a relationship between growth hormone levels and decreased CYP2C19 activity in these patients.

Thus currently, the mediators of the decreased activity of CYP2C19 in advanced cancer are not apparent. However, there was a significant relationship between body mass and CYP2C19 PM status. Although BMI is not a good indicator of cachexia particularly in the late stage disease, as weight loss due to increased muscle and fat catabolism, may be obscured by weight gain due to ascites. Nonetheless, the relationship between low BMI and poor metabolic status is intriguing. Cachexia is associated with increased proinflammatory cytokines particularly IL-6 and TNF-*α*, increased CRP, decreased albumin production and also decreased GH levels (Dülger *et al*, 2004) and hence may be a more general biomarker of the inflammatory status in these patients. Alternatively, the overall increased protein catabolism in cachexia may play a more direct role in the compromised CYP2C19 activity. The preliminary finding of a possible association between compromised CYP2C19 activity and low BMI requires further study in a larger cohort of patients.

The implications of compromised CYP2C19 activity are of direct relevance to cancer chemotherapy. The decrease in enzyme activity may significantly impact on the efficacy and toxicity of chemotherapeutic agents that are substrates for this enzyme. Moreover, a pharmacogenomic approach is often advocated to ‘personalise’ medicine to reduce the incidence of under- and over-dosing of patients. However, it is apparent that in cancer patients this approach would considerably underestimate the number of PM of drugs that are substrates for this enzyme.

## Figures and Tables

**Table 1:** The predicted CYP2C19 metabolic status (genotype) *vs* actual CYP2C19 activity (phenotype) in cancer patients

**Genotype**		**Phenotype**	
**Alleles**	**Predicted metaboliser status**	**Extensive metaboliser (log OMP HI<1)**	**Poor metaboliser (log OMP HI>1)**
*wt/wt* or *wt/var*	Extensive metaboliser	19	11
*var/var*	Poor metaboliser	0	1

There was a significant discordance between CYP2C19 genotype and phenotype (McNemar's test *P*<0.0005).

Two patients with *wt/wt* genotype had no detectable drug or metabolite in plasma and hence could not be phenotyped and were not included in this comparison.

**Table 2:** Comparison of the phenotypic poor metabolisers and extensive metabolisers

	**Phenotype**		
	**Poor metabolisers (log OMP HI >1)**	**Extensive metabolisers (log OMP HI <1)**	
	**median (IQ range) *n*=12**	**median (IQ range) *n*=19**	***P*-value^a^**
IL-1*α*	111.2 (17.7, 287.3)	107.2 (20.8, 794.5)	0.61
IL-1*β*	1.4 (0, 39.2)	1.1 (0, 2.3)	0.35
IL-6	65.9 (12.0, 82.7)	38.0 (13.4, 134.9)	0.97
TNF	7.4 (3.8, 12.5)	6.5 (4, 9.3)	0.57
TGF	497.5 (338.8, 641.3)	490 (440, 553)	0.63
CRP	9.1 (2.3, 39.3)	8.7 (2.0, 24.0)	0.76
Albumin	46.9 (43.4, 50.2)	44.8 (43.0, 48.9)	0.41
Growth hormone	988.8 (222.5, 3075)	955.7 (496.3, 4117.2)	0.44
BMI	27.7 (21.7, 30.0)	23.7 (22.0, 27.6)	0.09

Median and interquartile (IQ) ranges are shown for levels of inflammatory cytokines, albumin concentration, growth hormone concentration and body mass index (BMI).

a Mann–Whitney rank-sum test.
